# Fetal *in vivo* continuous cardiovascular function during chronic hypoxia

**DOI:** 10.1113/JP271091

**Published:** 2016-02-29

**Authors:** B. J. Allison, K. L. Brain, Y. Niu, A. D. Kane, E. A. Herrera, A. S. Thakor, K. J. Botting, C. M. Cross, N. Itani, K. L. Skeffington, C. Beck, D. A. Giussani

**Affiliations:** ^1^ Department of Physiology, Development & NeuroscienceUniversity of CambridgeDowning StreetCambridgeCB2 3EGUK; ^2^ Laboratorio de Función y Reactividad Vascular, Programa de Fisiopatología, Instituto de Ciencias BiomédicasFacultad de MedicinaUniversidad de Chile, SantiagoChile; ^3^ Department of RadiologyStanford University Medical CentrePalo AltoCA94305USA

## Abstract

**Key points:**

- The *in vivo* fetal cardiovascular defence to chronic hypoxia has remained by and large an enigma because no technology has been available to induce significant and prolonged fetal hypoxia whilst recording longitudinal changes in fetal regional blood flow as the hypoxic pregnancy is developing.
- We introduce a new technique able to maintain chronically instrumented maternal and fetal sheep preparations under isobaric chronic hypoxia for most of gestation, beyond levels that can be achieved by high altitude and of relevance in magnitude to the human intrauterine growth‐restricted fetus.
- This technology permits wireless recording in free‐moving animals of longitudinal maternal and fetal cardiovascular function, including beat‐to‐beat alterations in pressure and blood flow signals in regional circulations.
- The relevance and utility of the technique is presented by testing the hypotheses that the fetal circulatory brain sparing response persists during chronic fetal hypoxia and that an increase in reactive oxygen species in the fetal circulation is an involved mechanism.

**Abstract:**

Although the fetal cardiovascular defence to acute hypoxia and the physiology underlying it have been established for decades, how the fetal cardiovascular system responds to chronic hypoxia has been comparatively understudied. We designed and created isobaric hypoxic chambers able to maintain pregnant sheep for prolonged periods of gestation under controlled significant (10% O_2_) hypoxia, yielding fetal mean $P_{aO_{2}}$ levels (11.5 ± 0.6 mmHg) similar to those measured in human fetuses of hypoxic pregnancy. We also created a wireless data acquisition system able to record fetal blood flow signals in addition to fetal blood pressure and heart rate from free moving ewes as the hypoxic pregnancy is developing. We determined *in vivo* longitudinal changes in fetal cardiovascular function including parallel measurement of fetal carotid and femoral blood flow and oxygen and glucose delivery during the last third of gestation. The ratio of oxygen (from 2.7 ± 0.2 to 3.8 ± 0.8; *P* < 0.05) and of glucose (from 2.3 ± 0.1 to 3.3 ± 0.6; *P* < 0.05) delivery to the fetal carotid, relative to the fetal femoral circulation, increased during and shortly after the period of chronic hypoxia. In contrast, oxygen and glucose delivery remained unchanged from baseline in normoxic fetuses. Fetal plasma urate concentration increased significantly during chronic hypoxia but not during normoxia (Δ: 4.8 ± 1.6 *vs*. 0.5 ± 1.4 μmol l^−1^, P<0.05). The data support the hypotheses tested and show persisting redistribution of substrate delivery away from peripheral and towards essential circulations in the chronically hypoxic fetus, associated with increases in xanthine oxidase‐derived reactive oxygen species.

## Key points

- The *in vivo* fetal cardiovascular defence to chronic hypoxia has remained by and large an enigma because no technology has been available to induce significant and prolonged fetal hypoxia whilst recording longitudinal changes in fetal regional blood flow as the hypoxic pregnancy is developing.
- We introduce a new technique able to maintain chronically instrumented maternal and fetal sheep preparations under isobaric chronic hypoxia for most of gestation, beyond levels that can be achieved by high altitude and of relevance in magnitude to the human intrauterine growth‐restricted fetus.
- This technology permits wireless recording in free‐moving animals of longitudinal maternal and fetal cardiovascular function, including beat‐to‐beat alterations in pressure and blood flow signals in regional circulations.
- The relevance and utility of the technique is presented by testing the hypotheses that the fetal circulatory brain sparing response persists during chronic fetal hypoxia and that an increase in reactive oxygen species in the fetal circulation is an involved mechanism.

**Abbreviations**
HIF: hypoxia‐inducible factor
IUGR: intrauterine growth restriction
NO: nitric oxide
•O_2_^−^: superoxide anion
ROS: reactive oxygen species
XO: xanthine oxidase

## Introduction

The phrase ‘Everest *in utero*’ was coined by Sir Joseph Barcroft to highlight that the fetus develops under conditions of relative hypoxia compared with the oxygenation of the adult individual (Barcroft *et al*. 1933). Low oxygen tension in fetal life is essential for normal placental development as well as appropriate formation and maturation of the fetal cardiovascular system (Compernolle *et al*. 2003; Burton, 2009). However, reductions in fetal oxygenation below baseline can be harmful to the developing fetus unless appropriate compensatory responses are triggered. Short term, acute episodes of fetal hypoxia are common in late gestation, such as those occurring during transient compression of the umbilical cord (Giussani *et al*. 1997) or those resulting from myometrial contractions during labour and delivery (Huch *et al*. 1977). A sustained reduction from baseline in fetal oxygenation or chronic fetal hypoxia is associated with conditions of increased placental vascular resistance, leading to impaired uteroplacental blood flow. Chronic fetal hypoxia is therefore associated with pre‐eclampsia (Kingdom & Kaufmann, 1997), placental insufficiency (Pardi *et al*. 1993), chorioamnionitis (Maberry *et al*. 1990), gestational diabetes (Escobar *et al*. 2013) and even maternal obesity (Hayes *et al*. 2012; Kaplan‐Sturk *et al*. 2013). Chronic fetal hypoxia may also occur during impaired maternal oxygenation, as in maternal smoking (Longo, 1976), maternal respiratory diseases (Katz & Scheiner, 2008), maternal severe anaemia (Davis *et al*. 2005) or pregnancy at high altitude (Makowski *et al*. 1968; Giussani *et al*. 2001; Tissot van Patot *et al*. 2012; Soria *et al*. 2013). Acute episodes of severe fetal hypoxia may result in marked fetal acidosis and cardiovascular compromise with subsequent hypoxic–ischaemic encephalopathy, which is predictive of developing cerebral palsy and cognitive disability later in life (Low *et al*. 1985; Gunn & Bennet, 2009). Chronic fetal hypoxia can lead to fetal growth restriction, compromise the development of key organs and systems, and trigger an increased susceptibility to disease in later life (see Giussani & Davidge, 2013 for a review). Therefore, the fetal defence responses to acute and chronic hypoxia are essential to protect against significant morbidity and mortality in offspring. Not surprisingly, elucidation of the fetal compensatory responses to acute and chronic hypoxia and the mechanisms mediating them remains at the forefront of perinatal science and obstetric practice today.

The sheep fetus has long been the experimental model of choice for investigating fetal hypoxia *in vivo*. The fetal defence to acute hypoxia is contingent on the fetal cardiovascular system (Rudolph, 1984; Giussani *et al*. 1994). The fetal cardiovascular defence to acute hypoxia and the physiology underlying it are well established and characterised (see Giussani, 2015 for a review). In response to acute hypoxia, the fetus elicits bradycardia and redistributes its cardiac output (Cohn *et al*. 1974). Parallel measurement of continuous changes in carotid and femoral blood flow show cerebral vasodilatation and peripheral vasoconstriction, demonstrating *in vivo* the haemodynamics of the fetal brain sparing effect (Giussani *et al*. 1993). The bradycardia and peripheral vasoconstriction are triggered exclusively by a carotid chemoreflex (Giussani *et al*. 1993; Bartelds *et al*. 1993). The peripheral vasoconstriction is then maintained by the release of constrictor hormones into the fetal circulation (Jones & Robinson, 1975; Fletcher *et al*. 2006) as well as alterations in local factors, including the generation of reactive oxygen species (ROS) (Thakor *et al*. 2010, 2015; Kane *et al*. 2012, 2014). Therefore, the physiology underlying the fetal cardiovascular defence to acute episodes of hypoxia involves carotid chemoreflex and endocrine components as well as a local oxidant tone acting at the level of the fetal vasculature (see Giussani, 2016).

In marked contrast, the fetal *in vivo* haemodynamic responses to chronic fetal hypoxia and the mechanisms mediating them are not well characterised or understood. Progress in this field has been hampered in part by the inability to record continuous cardiovascular function in the fetus, including measurement of regional blood flow, as the chronic fetal hypoxia is actually occurring. Therefore, the objectives of this work were to introduce to the field a new technique for physiological research and to show the utility of the technique by investigating the fetal *in vivo* haemodynamic responses to significant chronic hypoxia in real time. We aimed to design and create isobaric hypoxic chambers able to maintain pregnant sheep for prolonged periods of gestation under controlled long‐term hypoxia, yielding fetal $P_{aO_{2}}$ levels similar to those measured in human intrauterine growth restriction (IUGR) pregnancy. Our second aim was to establish a wireless data acquisition system able to record fetal blood flow signals in addition to fetal blood pressure and fetal heart rate from free moving ewes as the hypoxic pregnancy was developing. Third, we wanted to use this system to determine *in vivo* in real time fetal cardiovascular function, including parallel measurement of fetal carotid and femoral blood flow and regional oxygen and glucose delivery during long‐term significant hypoxia in late gestation fetal sheep. We propose that alterations in the fetal vascular oxidant tone contribute to the fetal redistribution of blood flow away from the periphery during chronic fetal hypoxia. Therefore, the fourth aim of this work was to test the inter‐related hypotheses that the fetal circulatory brain sparing response does persist during significant chronic fetal hypoxia and that an increase in ROS in the fetal circulation is an involved mechanism. *In vivo* generation of ROS in the maternal and the fetal circulation was determined by measurement of changes in plasma urate and ascorbate concentrations, two of the few accepted biomarkers of ROS generation within the circulation *in vivo* (Halliwell & Gutteridge, 2004).

## Methods

### Ethical approval

All procedures were performed under the UK Animals (Scientific Procedures) Act 1986 and were approved by the Ethical Review Committee of the University of Cambridge.

### Surgical preparation, connection to CamDAS and post‐operative care

Twelve Welsh Mountain pregnant ewes and their singleton fetuses were surgically instrumented using strict aseptic techniques at 116 ± 1 days of gestational age (term is approximately 145 days), as described in detail (Fletcher *et al*. 2000, 2006). In brief, food but not water was withheld from the pregnant ewe for 24 h prior to surgery. On the day of surgery, the ewe was transferred to the preoperative room, where the neck fleece was clipped and anaesthesia was induced by injection of Alfaxan (1.5–2.5 mg kg^−1^ alfaxalone; Jurox Ltd, Worcestershire, UK) into the jugular vein. The ewe was then placed on her back and intubated (Portex cuffed endotracheal tube; Smiths Medical International Ltd, Ashford, UK) with the aid of a laryngoscope. Pre‐operative anaesthesia was maintained by spontaneous inhalation of 1.5% isoflurane in O_2_ (2 l min^−1^; IsoFlo; Abbott Laboratories Ltd, Maidenhead, UK) and the abdomen, flanks and medial surfaces of the hind limbs were shaved and cleaned.

The ewe was then transferred to the surgical suite operating table and the shaved and cleaned surfaces were scrubbed with alcohol in water, followed by a spray of hibitane solution (Hibitane Plus in alcohol and water; 5% chlorohexidine gluconate; Regent Medical Ltd, Manchester, UK) and another spray of concentrated iodine solution (Povidone‐Iodine; Seton Healthcare Group PLC, Oldham, UK). General anaesthesia (1.5–2.0% isoflurane in 60:40 O_2_/N_2_O) was maintained using positive pressure ventilation in a non‐rebreathing circuit (Datex‐Ohmeda Ltd, Hatfield, UK). Antibiotics (30 mg kg^−1^ i.m. procaine benzylpenicillin; Depocillin; Intervet UK Ltd, Milton Keynes, UK) and an analgesic agent (1.4 mg kg^−1^ s.c. carprofen; Rimadyl; Pfizer Ltd, Sandwich, UK) were administered immediately before the start of surgery. The animal was covered with a plastic sterile drape (Buster Opcover; Buster, Kruuse, Denmark) and with sterile surgical linen drapes on top, such that only the midline incision site was left exposed. Midline abdominal and uterine incisions were then made, the fetal hind limbs were exteriorised minimising amniotic fluid loss and, on one side, fetal femoral arterial (i.d. 0.86 mm, o.d. 1.52 mm; Critchly Electrical Products, Kingsgrove, NSW, Australia) and venous (i.d. 0.56 mm, o.d. 0.96 mm) catheters were inserted. The catheter tips were advanced to the descending aorta and inferior vena cava, respectively. Another catheter was anchored onto the fetal hind limb for recording of the reference amniotic pressure. A Transonic flow probe was positioned around the contralateral femoral artery (MC2RS‐JSF‐WC120‐CS12‐GCP, Transonic Systems, Ithaca, NY, USA). The fetal skin incisions were closed with thin linen suture and the uterine incision was closed in layers (3‐0 Dexon II Bi‐colour; Sherwood, Davis & Geck, Gosport, UK). The dead space of the catheters was filled with heparinised saline (80 i.u. heparin ml^−1^ in 0.9% NaCl) and the catheter ends were plugged with sterile brass pins. The fetal head was then palpated and exteriorised through a second uterine incision. The fetal carotid arteries were isolated and on one side a catheter was inserted with the tip remaining in the ascending aorta. A second Transonic flow probe (MC2RS‐JSF‐WC120‐CS12‐GCP) was positioned around the contralateral carotid artery (Giussani *et al*. 1993) and the fetal skin incision and the second uterine incision were closed as before. All catheters were then exteriorised via a keyhole incision in the maternal flank on the ewe's right side whilst the flow probe leads were exteriorised through a keyhole incision on the ewe's left flank. The maternal peritoneum was then closed in three segments with thick linen suture, and the maternal abdominal skin incision was sewn together (Ethilon 2‐0; Ethicon Ltd, Edinburgh, UK). A Teflon catheter (i.d. 1.0 mm, o.d. 1.6 mm; Altec, St Austell, UK) was then inserted into the maternal femoral artery and placed in the descending aorta, and a maternal venous catheter placed in the inferior vena cava (i.d. 0.86 mm, o.d. 1.52 mm; Critchly Electrical Products). These catheters were exteriorised through the same keyhole on the ewe's right side flank.

**Figure 1:**
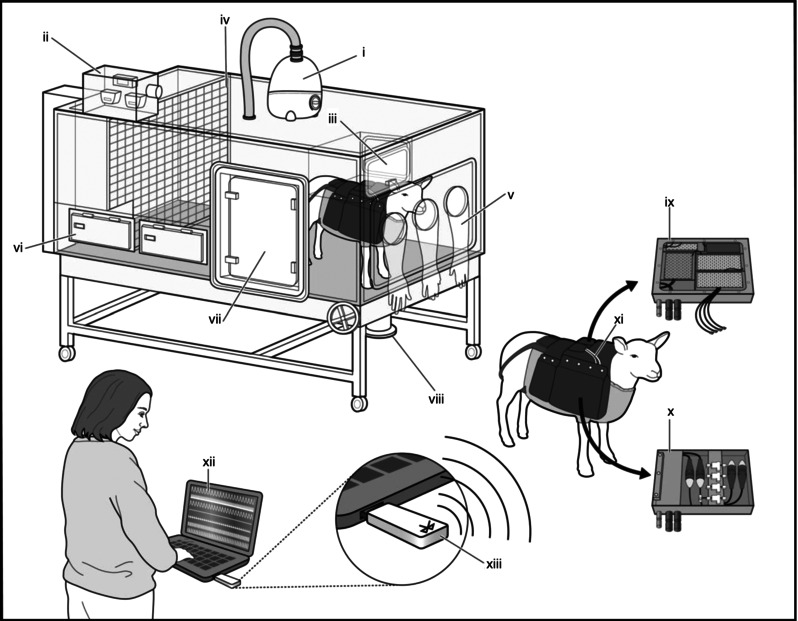
**Isobaric hypoxic chambers and the CamDAS system** Each chamber was equipped with an electronic servo‐controlled humidity cool steam injection system to return the appropriate humidity to the inspirate (i). Ambient $P_{O_{2}}$, $P_{CO_{2}}$, humidity and temperature within each chamber were monitored via sensors (ii). For experimental procedures, each chamber had a double transfer port (iii) to internalise material and a manually operated sliding panel (iv) to bring the ewe into a position where daily sampling of blood could be achieved through glove compartments (v). Each chamber incorporated a drinking bowl on continuous water supply and a rotating food compartment (vi) for determining food intake. A sealed transfer isolation cart could be attached to a side exit (vii) to couple chambers together for cleaning. Waste was disposed off via a sealable waste pipe (viii). The CamDAS system was contained in a custom‐made sheep jacket able to hold a box containing the Transonic flow probe connectors on one side (ix) and the pressure acquisition system box (x) on the other. Cables (xi) connected the two boxes together and also linked to two battery packs able to power the system for 24 hours. Measurements made using the data acquisition were transmitted wirelessly to a laptop kept outside the chamber room (xii) via Bluetooth (xiii), thereby making it possible to view continuous recordings of the maternal and fetal cardiovascular data.

A custom made jacket designed to house the bespoke wireless Cambridge Data Acquisition System (CamDAS, Maastricht Instruments, Maastricht, The Netherlands) was then fitted to the ewe. The CamDAS contained a pressure box and a flow box able to record simultaneously four pressure and four flow signals, respectively (Fig. 1). It was powered by lithium batteries which were also housed within the jacket. The catheters were then connected to pressure transducers (COBE; Argon Division, Maxxim Medical, Athens, TX, USA) within the pressure box and the flow probes were connected to the flow box. Heart rate was triggered from the blood pressure and flow waveforms. Recordings of fetal arterial blood pressure and fetal heart rate, amniotic pressure, fetal carotid blood flow and fetal femoral blood flow could then be continuously transmitted wirelessly via Bluetooth technology onto a laptop computer. At this time the anaesthetic was turned off and the ewe was ventilated until spontaneous respiratory movements were observed. The ewe was extubated when spontaneous breathing returned and the animal was allowed to recover in a floor pen with free access to food and water.

Ewes wearing jackets with the CamDAS were housed in individual pens in rooms with a 12:12 h light–dark cycle where they had free access to hay and water and were fed concentrates twice daily (100 g sheep nuts no. 6; H & C Beart Ltd, Kings Lynn, UK). Antibiotics were administered daily to the ewe (0.20–0.25 mg kg^−1^ i.m. depocillin; Mycofarm, Cambridge, UK) for the first 3 days of recovery and daily to the fetus i.v. and into the amniotic cavity (600 mg in 2 ml 0.9% NaCl, benzylpenicillin; Crystapen, Schering‐Plough, Animal Health Division, Welwyn Garden City, UK). Generally, normal feeding patterns were restored within 24–48 h of recovery. Ewes were then randomly allocated to one of two experimental groups: normoxia (*n* = 6) or chronic hypoxia (*n* = 6).

### Chronic hypoxia protocol

Ewes allocated to chronic hypoxia were housed in one of four bespoke isobaric hypoxic chambers (Telstar Ace, Dewsbury, UK; Fig. 1). These chambers were supplied with variable amounts of nitrogen and air provided via nitrogen generators and air compressors, respectively, from a custom designed nitrogen generating system (Domnick Hunter Gas Generation, Gateshead, UK). The system operated continuously, automatically switching between adsorption beds of two nitrogen generators (Domnick Hunter N2MAX112X2) to ensure a constant provision of pure nitrogen gas. The purity of the nitrogen was monitored to ensure only gas of the required purity reached the application. Compressed air and compressed nitrogen were then piped to the laboratory containing the hypoxic chambers and gases were blended to requirements. The inspirate air mixture underwent a minimum of 12 changes per hour in each chamber and the incoming air mixture was passed via silencers able to reduce noise levels within the hypoxic chamber laboratory (76 dB(A)) and inside each chamber (63 dB(A)) to values lower than those necessary to abide by the Control of Noise at Work Regulations. This not only complied with human health and safety and animal welfare regulations but also provided a tranquil environment for the animal inside each chamber. All chambers were equipped with an electronic automatic humidity cool steam injection system (1100‐03239 HS‐SINF; Masalles, Barcelona, Spain) to ensure appropriate humidity in the inspirate (55 ± 10%). Ambient PO_2_, $P_{CO_{2}}$, humidity and temperature within each chamber were monitored via sensors, displayed and values recorded continuously via the Trends Building Management System of the University of Cambridge through a secure Redcare intranet. In this way, the percentage of oxygen in the isolators could be controlled with precision continuously over long periods of time. For experimental procedures, each chamber had a double transfer port to internalise material and a manually operated sliding panel to encourage the ewe into a position where daily sampling of blood could be achieved through glove compartments (Fig. 1). Each chamber incorporated a drinking bowl on continuous water supply and a rotating food compartment which could be removed for determining food intake. The chambers were transparent, allowing ewes to visualise each other. A transfer isolation cart could couple two chambers together, allowing ewes to move transiently to an adjacent chamber maintained at the same oxygen environment. This was necessary for cleaning the chambers, which occurred once per week. Therefore, all experimental and maintenance procedures could be carried out without interruption of the hypoxic exposure.

Pregnancies assigned to the chronic hypoxia group were placed inside the chambers under normoxic conditions (11 l s ^−1^ air, equating to 39.6 m^3^ h^−1^) for 2 days. On the fifth post‐operative day at 121 ± 1 days of gestation, pregnancies assigned to chronic hypoxia were exposed to *ca* 10% O_2_ by altering the incoming gas mixture to 5 l s^−1^ air/6 l s^−1^ N_2_. The induction of hypoxia was gradual, achieving 10% O_2_ over 24 h. Following 11 days of chronic hypoxia exposure, the pregnancies were returned to breathing normoxic air again within the chambers. Cardiovascular data were transmitted wirelessly via Bluetooth technology and recorded onto a laptop kept outside the hypoxic chamber laboratory. This permitted continuous *in vivo* recordings of the maternal and fetal cardiovascular data without disturbing the animal's environment.

Pregnancies allocated to the normoxia group were housed in a barn in floor pens with the same floor area as that of the hypoxic chambers. Both the normoxia and the chronic hypoxia groups of ewes were fed daily the same bespoke maintenance diet made up of concentrate pellets and hay (40 g nuts kg^–1^ and 3 g hay kg^–1^; Manor Farm Feeds Ltd, Oakham, UK) to facilitate the monitoring of food intake.

### Blood sampling regimen and analysis

Samples (0.3 ml) of ascending and descending aortic fetal as well as descending aortic maternal blood were taken daily for measurement of fetal and maternal blood gas, acid base and metabolic status. Arterial blood gas and acid base values were measured using an ABL5 blood gas analyser (Radiometer, Copenhagen, Denmark; maternal measurements corrected to 38 °C, fetal measurements corrected to 39.5 °C). Values for percentage saturation of haemoglobin with oxygen (SatHb) and for the concentration of haemoglobin in blood ([Hb]) were determined using a haemoximeter (OSM3; Radiometer). Blood glucose and lactate concentrations were measured using an automated analyser (Yellow Springs 2300 Stat Plus Glucose/Lactate Analyser; YSI Ltd, Farnborough, UK). Values for haematocrit were obtained in duplicate using a microhaematocrit centrifuge (Hawksley, Lancing, UK). An additional 1 ml of maternal arterial and fetal arterial blood was taken during baseline (24 h prior to chronic hypoxia) and at +1 h, +6 h, +1 day, +5 days and +10 days of chronic hypoxia and at +2 days following chronic hypoxia or at equivalent times in normoxic animals for determination of plasma vitamin C and urate concentrations.

### Determination of plasma urate and vitamin C

Plasma concentrations of urate were measured using an automated Siemens Dimension RxL analyser (Dimension RxL Max integrated Chemistry system, Siemens, UK, Core Biochemical Assay Laboratory, Cambridge, UK). In brief, urate in the plasma (taken from previously unthawed heparin‐treated sample aliquots) is converted to allantoin by the action of uricase (urate oxidase). As urate is able to absorb light at 293 nm but allantoin is not, the change in absorbance at 293 nm is directly proportional to the urate concentration in the sample. Additional dilutions of low calibrator solutions were used to improve the reproducibility of low analyte concentrations. The inter‐assay coefficients of variation were 5.0% at 200 μmol l^−1^ and 2.7% at 560 μmol l^−1^. The lower limit of detection of the assay was 6 μmol l^−1^.

Plasma concentrations of ascorbic acid were measured by a fluorimetric technique using a centrifugal analyser with a fluorescence attachment, according to the method of Vuilleumier & Keck (1989; Core Biochemical Assay Laboratory, Cambridge, UK). In brief, aliquots of maternal and fetal plasma (acidified 1:1 with ice‐cold 10% metaphosphoric acid) were centrifuged and the supernatant stored at −80 °C. They were then loaded in duplicate onto a black microtitre plate with standards and quality controls. Addition of ascorbate oxidase converts any vitamin C in the sample to dehydroascorbic acid, which is then condensed with 1,2‐phenyldiamine to form a fluorescent quinoxaline derivative. The fluorescence is measured on a Fluoroskan Ascent FL (Fluoroskan Ascent FL Microplate Fluorometer and Luminometer, Thermo Fisher Scientific, Basingstoke, UK) and is proportional to the vitamin C concentration in the sample. The inter‐assay coefficients of variation were 7.9% at 27.1 μmol l^−1^ and 5.0% at 89.7 μmol l^−1^; the lower limit of detection of the assay was 10 μmol l^−1^.

### Data and statistical analyses

All data are expressed as mean ± SEM. For the cardiovascular data, minute by minute average values were downloaded continuously throughout the experiment and imported into an Excel spreadsheet.

Values during 2 h morning (10.00–12.00 h) and night‐time (22.00–24.00 h) epochs were then averaged for each day. Fetal arterial blood oxygen content (O_2_ content) was calculated using eqn (1):

(1) O2 content ( mmol .l−1)= Hb × SatHb 100×0.62

where [Hb] (g dl^−1^) is the blood concentration of haemoglobin, SatHb is the percentage oxygen saturation of haemoglobin and where 1 molecule of Hb (mol. wt 64 450) binds 4 molecules of oxygen. The contribution of oxygen dissolved in plasma is regarded as negligible (Owens *et al*. 1987). Values for oxygen and for glucose delivery to the fetal ascending and descending aorta were then calculated using eqns (2) and (3), respectively:

(2) Oxygen  delivery (μ mol .min−1)=O2 content (μ mol .ml−1)× appropriate  flow ( ml . mi n−1)

(3) Glucose  delivery (μ mol .min−1)= Glucose (μ mol .ml−1)× appropriate  flow ( ml .min−1)

For statistical analysis, cardiovascular data were analysed comparing the effect of treatment, time and interactions between treatment and time using two‐way repeated measures ANOVA with Tukey's *post hoc* test. Where relevant, area under the curve or slopes were analysed to better summarise the data. Comparison of slopes was performed using the Student's *t* test for unpaired data. For all comparisons, statistical significance was accepted at *P* < 0.05.

## Results

### Maternal food consumption, arterial blood gas, acid base and metabolic status

**Figure 2:**
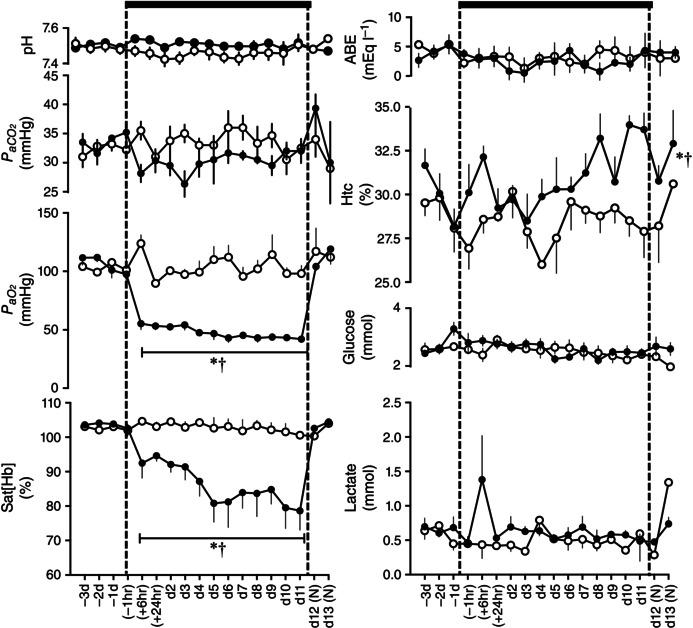
**Maternal blood gas, acid base and metabolic status** Values are mean ± SEM for pregnant sheep undergoing normoxic (**◯**, *n* = 6) or chronic hypoxic (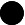, *n* = 6) pregnancy. Maternal blood gas values were corrected to 38 °C. pH, arterial pH; P aC O2, arterial CO_2_ partial pressure; $P_{aO_{2}}$, arterial O_2_ partial pressure; Sat[Hb], percentage saturation of haemoglobin; ABE, acid base excess; Htc, haematocrit; Glucose, blood glucose concentration; Lactate, blood lactate concentration; (N), normoxic recovery. The x‐axis shows time in hours (hr) and days (d). Significant differences (*P* < 0.05): *differences indicating a significant main effect of time compared with baseline; ^†^differences indicating a significant main effect of treatment compared with normoxic pregnancy (two‐way repeated‐measures ANOVA + Tukey test). For Htc, the comparison of slopes was achieved with the Student's *t* test for unpaired data.

Basal maternal daily food consumption was not different between groups (normoxic (N): 1.3 ± 0.9 *vs*. hypoxic (H): 1.1 ± 0.4 kg day^−1^). Exposure to chronic hypoxia did not affect maternal daily food intake (N: 1.5 ± 0.9 *vs*. H: 1.5 ± 0.9 kg day^−1^). Basal values for maternal arterial blood gas, acid base and metabolic status were not different between groups and were within the normal range for Welsh Mountain ewes at the appropriate time of gestation prior to experimentation (Fletcher *et al*. 2002, 2006 Fig. 2). Ewes exposed to chronic hypoxia had a significant reduction in the partial pressure of arterial oxygen (105.7 ± 3.7 to 42.0 ± 1.2 mmHg) and in oxygen saturation (103.5 ± 0.5 to 78.6 ± 5.7%) compared to controls ($P_{aO_{2}}$: 104.2 ± 1.9 and Sat[Hb]: 92.36 ± 1.5) and their own baseline (*P* < 0.05, Fig. 2). Furthermore, ewes exposed to chronic hypoxia had significantly elevated haematocrit by the end of exposure relative to controls (33.9 ± 1.0 *vs*. 28.5 ± 0.9%). These changes occurred without significant alteration from baseline or between groups in arterial pH, partial pressure of arterial carbon dioxide, acid base excess, blood glucose or blood lactate concentrations (Fig. 2). Maternal blood lactate concentrations showed a transient 24 h increase following the onset of hypoxia, but these alterations did not reach significance. While values for partial pressure of arterial oxygen and oxygen saturation returned towards basal levels, values for haematocrit remained significantly elevated in the chronic hypoxia ewes following re‐oxygenation.

### Fetal arterial blood gas, acid base and metabolic status

**Figure 3:**
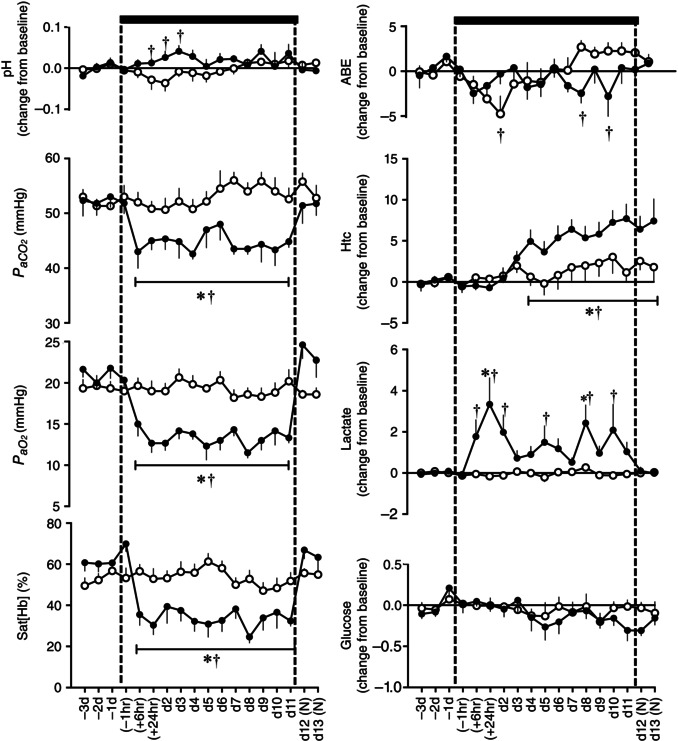
**Fetal blood gas, acid base and metabolic status** Values are mean ± SEM for fetal sheep undergoing normoxic (**◯**, *n* = 6) or chronic hypoxic (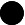, *n* = 6) pregnancy. Fetal blood gas values were corrected to 39.5 °C. pH, arterial pH; P aC O2, arterial CO_2_ partial pressure; $P_{aO_{2}}$, arterial O_2_ partial pressure; Sat[Hb], percentage saturation of haemoglobin; ABE, acid base excess; Htc, haematocrit; Glucose, blood glucose concentration; Lactate, blood lactate concentration; (N), normoxic recovery. The x‐axis shows time in hours (hr) and days (d). Significant differences (*P* < 0.05): *differences indicating a significant main effect of time compared with baseline; ^†^differences indicating a significant main effect of treatment compared with normoxic pregnancy (two‐way repeated‐measures ANOVA + Tukey test).

Basal values for descending aortic fetal arterial blood gas, acid base and metabolic status were similar between groups and were within the normal range for Welsh Mountain singleton sheep fetuses at this stage of gestation (Fletcher *et al*. 2002, 2006, Fig. 3). Fetuses exposed to chronic hypoxia had a significant reduction from baseline in the partial pressure of arterial oxygen (20.9 ± 0.5 to 11.5 ± 0.6 mmHg) and oxygen saturation (63.0 ± 1.9 to 24.6 ± 2.9%, *P* < 0.05, Fig. 3). Fetuses exposed to chronic hypoxia also had significantly elevated haematocrit by the end of exposure relative to controls (36.1 ± 1.3 *vs*. 28.0 ± 0.5, *P* < 0.05). Chronically hypoxic fetuses also showed a transient increase in arterial pH and reductions in acid base excess by the end of the hypoxic period, and sustained falls in the partial pressure of arterial carbon dioxide and increases in blood lactate concentrations during the hypoxic exposure (Fig. 3). These effects occurred without significant alteration from baseline in blood glucose concentrations. While values for all altered variables returned towards baseline, values for haematocrit remained significantly elevated in the chronic hypoxia fetuses following re‐oxygenation.

### Fetal cardiovascular responses

**Figure 4:**
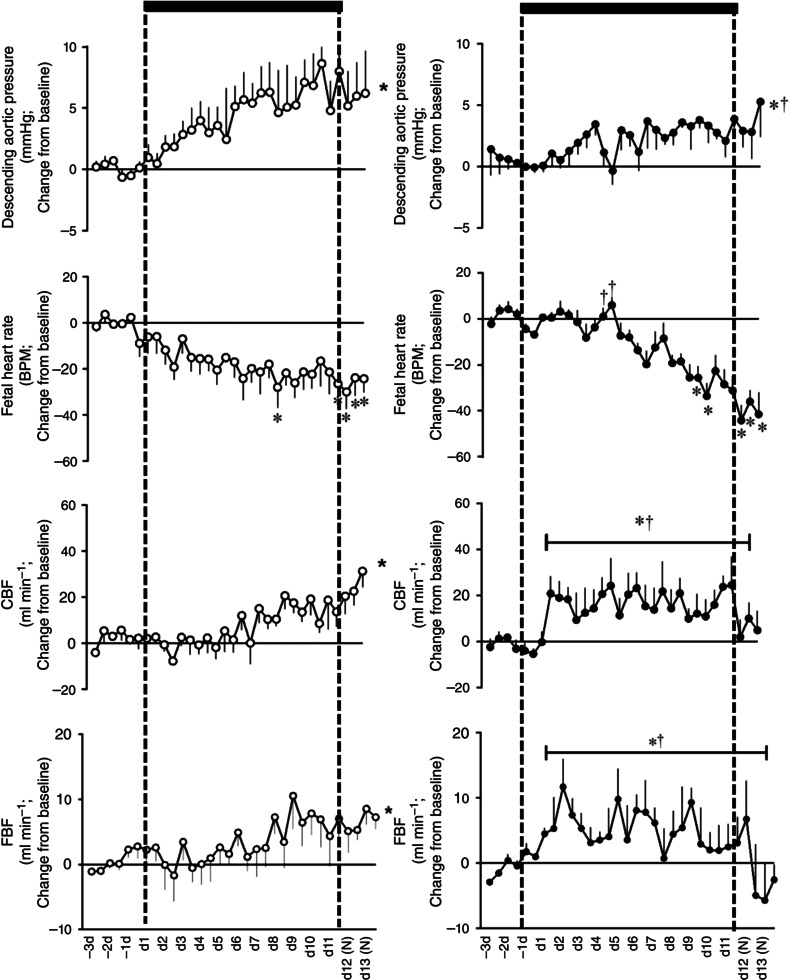
**Fetal cardiovascular responses to chronic hypoxia** Values are mean ± SEM for the change from baseline in cardiovascular variables in fetal sheep undergoing normoxic (**◯**, *n* = 6, left) or chronic hypoxic (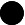, *n* = 6, right) pregnancy. CBF, carotid blood flow; FBF, femoral blood flow; BPM, beats per minute; (N), normoxic recovery. The x‐axis shows time in days (d). Significant differences (*P* < 0.05): *differences indicating a significant main effect of time compared with baseline; ^†^differences indicating a significant main effect of treatment compared with normoxic pregnancy (two‐way repeated‐measures ANOVA + Tukey test). For descending aortic pressure, the two‐way ANOVA represents a comparison of slopes. For FBF and CBF, the two‐way ANOVA represents a comparison of areas under the curve.

Basal values for fetal descending aortic blood pressure (41.1 ± 0.6 *vs*. 39.2 ± 0.5 mmHg), fetal heart rate (186.1 ± 2.0 *vs*. 177.3 ± 1.8 beats min^−1^), carotid blood flow (73.3 ± 3.0 *vs*. 75.7 ± 1.9 ml min^−1^) and femoral blood flow (32.3 ± 1.1 *vs*. 35.5 ± 1.5 ml min^−1^) were not different between normoxic and chronic hypoxic groups and were within the normal range for Welsh Mountain singleton sheep fetuses at this stage of gestation (Giussani *et al*. 1993; Jellyman *et al*. 2005, 2009). Fetuses undergoing normoxic pregnancy showed progressive increases in arterial blood pressure (41.1 ± 0.6 to 50.1 ± 2.3 mmHg), carotid blood flow (73.3 ± 3.0 to 92.3 ± 7.4 ml min^−1^) and femoral blood flow (32.3 ± 1.1 to 35.9 ± 2.5 ml min^−1^) and progressive decreases in heart rate (186.1 ± 2.0 to 163.3 ± 7.0 beats min^−1^) with advancing gestational age (*P* < 0.05, Fig. 4). In contrast, in fetuses exposed to chronic hypoxia, the increment in fetal arterial blood pressure with advancing gestation was significantly diminished and the decrement in fetal heart rate occurred much later following the onset of hypoxia but reaching similar levels by the end of the period of exposure (Fig. 4). Furthermore, fetuses exposed to chronic hypoxia showed sustained elevations in both carotid and femoral blood flow during exposure (Table 1 and Fig. 4). While values for carotid and femoral blood flow returned towards basal levels, values for arterial blood pressure and for heart rate remained significantly altered from baseline in the chronic hypoxia fetuses following re‐oxygenation.

**Table 1:** Variables used to calculate oxygen and glucose delivery in the chronically hypoxic fetus

	Descending aortic		Descending aortic		Ascending aortic		Ascending aortic					
	O_2_ content		blood glucose		O_2_ content		blood glucose		Carotid blood flow		Femoral blood flow	
	(mmol l ^−1^)		(mmol l^−1^)		(mmol l^−1^)		(mmol l^−1^)		(ml min^−1^)		(ml min^−1^)	
	N	H	N	H	N	H	N	H	N	H	N	H
Experi‐												
mental												
day												
−3	2.81 ± 0.08	3.20 ± 0.24	0.89 ± 0.17	0.86 ± 0.03	3.17 ± 0.13	3.90 ± 0.19	0.97 ± 0.26	0.91 ± 0.04	68.8 ± 13.8	69.7 ± 2.4	30.8 ± 4.6	32.0 ± 2.9
−2	2.93 ± 0.22	3.17 ± 0.19	0.90 ± 0.11	0.89 ± 0.08	3.23 ± 0.18	3.25 ± 0.05	0.83 ± 0.20	0.85 ± 0.05	71.7 ± 10.4	81.2 ± 4.0	29.6 ± 3.8	36.1 ± 3.4
−1	3.14 ± 0.12	3.30 ± 0.07	0.83 ± 0.15	1.20 ± 0.12	3.17 ± 0.14	3.59 ± 0.07	0.98 ± 0.12	1.17 ± 0.12	72.8 ± 9.1	74.3 ± 5.3	28.5 ± 0.6	36.1 ± 3.4
(−1 h)	2.89 ± 0.44	3.42 ± 0.20	0.72 ± 0.13	0.98 ± 0.11	3.17 ± 0.38	3.65 ± 0.22	0.83 ± 0.07	0.96 ± 0.11	69.7 ± 10.8	81.4 ± 7.1	35.9 ± 0.0	37.3 ± 1.9
(+6 h)	2.89 ± 0.41	1.82 ± 0.24*†	0.78 ± 0.10	1.02 ± 0.07	3.39 ± 0.47	1.91 ± 0.21*†	0.79 ± 0.09	1.04 ± 0.10	68.2 ± 12.3	104.1 ± 7.5*	35.5 ± 0.4	44.3 ± 4.1
(+24 h)	2.93 ± 0.50	1.63 ± 0.31*†	0.76 ± 0.16	0.97 ± 0.08	3.34 ± 0.43	1.89 ± 0.32*†	0.73 ± 0.10	0.98 ± 0.10	64.5 ± 8.9	96.8 ± 9.9	33.1 ± 1.2	47.1 ± 3.2*
2	2.81 ± 0.19	2.08 ± 0.38*	0.82 ± 0.14	0.92 ± 0.08	3.21 ± 0.15	2.01 ± 0.26*†	0.76 ± 0.11	0.86 ± 0.09	76.9 ± 11.8	90.9 ± 10.7†	35.8 ± 1.1	40.8 ± 3.0*†
3	3.05 ± 0.17	2.25 ± 0.37*	0.79 ± 0.09	1.03 ± 0.15	3.54 ± 0.15	2.51 ± 0.27*†	0.82 ± 0.10	0.96 ± 0.14	78.2 ± 8.2	86.2 ± 12.1†	33.8 ± 3.0	36.2 ± 1.1†
4	3.03 ± 0.22	2.17 ± 0.30*†	0.77 ± 0.18	0.98 ± 0.03	3.49 ± 0.19	2.56 ± 0.24*†	0.70 ± 0.15	0.89 ± 0.05	74.8 ± 7.7	104.4 ± 11.7*	36.0 ± 3.6	45.6 ± 3.6†
5	3.22 ± 0.41	1.89 ± 0.39*†	0.67 ± 0.16	0.84 ± 0.08	3.52 ± 0.20	2.01 ± 0.32*†	0.72 ± 0.12	0.79 ± 0.07	69.3 ± 6.0	96.4 ± 11.6*	35.4 ± 4.3	43.3 ± 1.5
6	3.01 ± 0.41	2.16 ± 0.35*	0.61 ± 0.09	0.92 ± 0.04	3.45 ± 0.29	2.44 ± 0.38*†	0.73 ± 0.09	0.96 ± 0.06	77.3 ± 7.1	88.2 ± 11.5*	35.9 ± 5.1	38.8 ± 2.3†
7	2.74 ± 0.25	2.64 ± 0.35*	0.72 ± 0.15	0.89 ± 0.08	3.03 ± 0.10	2.81 ± 0.35	0.73 ± 0.16	0.91 ± 0.11	82.2 ± 10.0	103.7 ± 14.6	38.9 ± 4.8	40.8 ± 1.9
8	3.20 ± 0.38	1.62 ± 0.17*†	0.82 ± 0.10	0.90 ± 0.11	3.33 ± 0.42	1.79 ± 0.12*†	0.77 ± 0.15	0.92 ± 0.11	81.9 ± 7.8	101.4 ± 7.7*	44.2 ± 1.2	43.2 ± 1.9
9	2.86 ± 0.09	2.27 ± 0.40*	0.80 ± 0.11	0.78 ± 0.11	3.29 ± 0.16	2.67 ± 0.20*	0.69 ± 0.13	0.84 ± 0.09	93.0 ± 12.7	82.4 ± 11.6*	45.4 ± 3.3*	35.7 ± 1.7
10	2.73 ± 0.11	2.56 ± 0.54*	0.72 ± 0.15	0.81 ± 0.07	3.40 ± 0.04	2.92 ± 0.61*	0.85 ± 0.16	0.84 ± 0.08	90.2 ± 10.1	94.9 ± 8.3	37.0 ± 1.2*	39.5 ± 1.8
11	3.11 ± 0.21	2.30 ± 0.16*†	0.62 ± 0.10	0.79 ± 0.04	3.26 ± 0.27	2.69 ± 0.29*	0.80 ± 0.15	0.82 ± 0.07	82.5 ± 5.4	101.3 ± 12.6*	36.2 ± 1.3	39.9 ± 7.2
12(N)	3.03 ± 0.20	4.40 ± 0.19†	0.69 ± 0.11	0.62 ± 0.04	3.35 ± 0.17	4.58 ± 0.18†	0.86 ± 0.17	0.65 ± 0.05	83.2 ± 10.3	85.3 ± 7.2*	36.8 ± 1.0	29.7 ± 3.7†
13(N)	2.64 ± 0.17	4.35 ± 0.38†	0.69 ± 0.12	0.80 ± 0.06	2.70 ± 0.12	4.82 ± 0.25	0.66 ± 0.13	0.76 ± 0.03	101.8 ± 16.2*	92.9 ± 9.7*	37.1 ± 2.3	30.1 ± 0.7

Values are mean ± SEM variables required to calculate oxygen and glucose delivery throughout the experimental protocol. Data are shown for fetal ascending and descending arterial oxygen content, blood glucose and flows in normoxic (N) and hypoxic (H) fetuses (*n* = 6 both groups). Significant differences (*P* < 0.05): *differences indicating a significant main effect of time compared with baseline; †differences indicating a significant main effect of treatment compared with normoxic pregnancy (two‐way repeated‐measures ANOVA + Tukey test).

### Fetal ascending and descending aortic oxygen and glucose delivery

**Figure 5:**
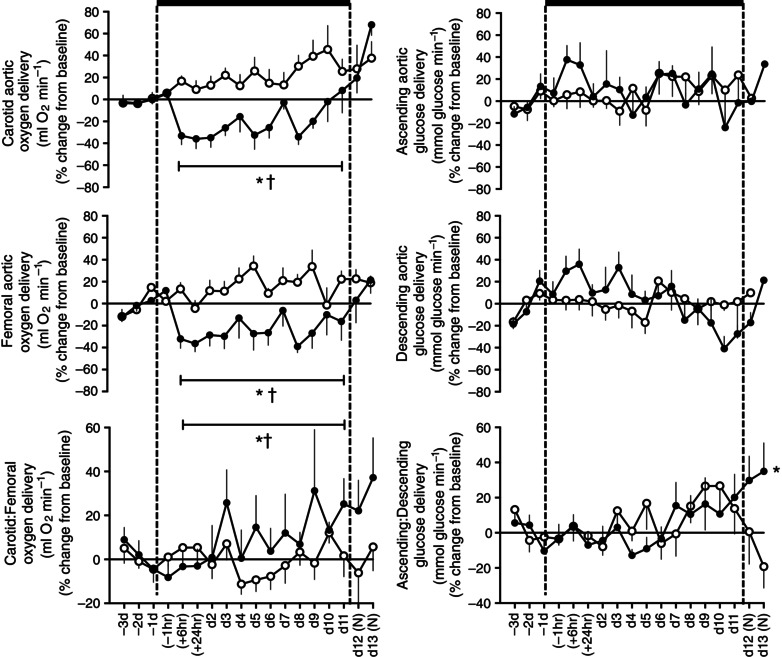
**Fetal carotid and femoral arterial oxygen and glucose delivery in the chronically hypoxic fetus** Values are mean ± SEM for the change from baseline in oxygen and glucose delivery in the ascending and the descending aorta and the ratio of these values in fetal sheep undergoing normoxic (**◯**, *n* = 6) or chronic hypoxic (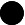, *n* = 6) pregnancy. (N), normoxic recovery. The x‐axis shows time in hours (hr) and days (d). Significant differences (*P* < 0.05): *differences indicating a significant main effect of time compared with baseline; ^†^differences indicating a significant main effect of treatment compared with normoxic pregnancy (two‐way repeated‐measures ANOVA + Tukey test). For the ratio of ascending/descending oxygen delivery, the two‐way ANOVA represents an analysis of the area under the curve.

Values for oxygen and glucose delivery to the ascending and descending aortic circulations were calculated using the values for oxygen content, blood glucose and blood flow in the relevant circulations shown in Table 1. Fetuses exposed to chronic hypoxia showed significantly reduced values for oxygen delivery to both ascending (227.0 ± 9.8 *vs*. 256.7 ± 7.9 μmol min^−1^) and descending (90.6 ± 4.7 *vs*. 110.7 ± 4.7 μmol min^−1^) aortic circulations relative to normoxic fetuses (Fig. 5). However, when oxygen delivery was expressed as a ratio between vascular beds, there was a significant increase in the oxygen delivery to the ascending relative to the descending aortic circulation during chronic hypoxia (*P* < 0.05, Fig. 5). In contrast, glucose delivery to either ascending or descending aorta was unaltered from baseline in fetuses exposed to chronic hypoxia. However, a significant increase in glucose delivery to the ascending aorta was calculated when expressed as a ratio relative to values for the descending aorta by the end of the experimental protocol (Fig. 5).

### Maternal and fetal plasma urate and vitamin C concentrations

**Figure 6:**
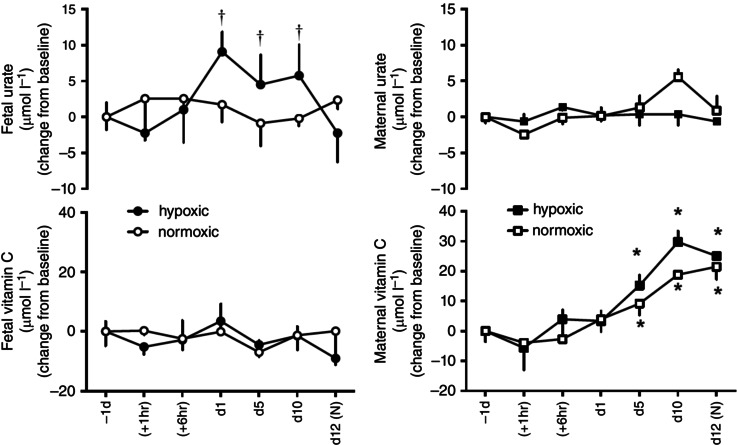
**Fetal and maternal vitamin C and urate levels in the chronically hypoxic fetus** Values are mean ± SEM for the change from baseline in vitamin C and urate in pregnant ewes and fetal sheep undergoing normoxic (**◯**, fetus; □, ewe; *n* = 6) or chronic hypoxic (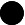, fetus; ■, ewe; *n* = 6) pregnancy. (N), normoxic recovery. The x‐axis shows time in hours (hr) and days (d). Significant differences (*P* < 0.05): *differences indicating a significant main effect of time compared with baseline; ^†^differences indicating a significant main effect of treatment compared with normoxic pregnancy (two‐way repeated‐measures ANOVA + Tukey test). For the fetal urate levels the two‐way ANOVA represents an analysis of the area under the curve.

Values for basal urate concentrations were significantly higher in the fetal (N: 25.5 ± 1.7 μmol l^−1^ and H: 21.3 ± 2.1 μmol l^−1^) than in the maternal (N: 7.5 ± 0.8 μmol l^−1^ and H: 6.6 ± 0.7 μmol l^−1^) circulation in both normoxic and hypoxic pregnancy (*P* < 0.05). While fetal plasma urate concentrations increased from baseline in fetuses undergoing chronic hypoxia, plasma urate remained unchanged from baseline in fetuses undergoing normoxic pregnancy and in mothers of normoxic or hypoxic pregnancy (Fig. 6). In contrast, values for basal vitamin C concentrations were similar in the fetal (N: 30.4 ± 4.8 μmol l^−1^ and H: 31.8 ± 3.5 μmol l^−1^) and in the maternal (N: 31.4 ± 4.2 μmol l^−1^ and H: 33.9 ± 1.1 μmol l^−1^) circulation in both normoxic and hypoxic pregnancy. However, while fetal levels of vitamin C remained unchanged from baseline, there was a progressive increase in maternal plasma vitamin C with advancing gestation in both normoxic and hypoxic pregnancy (Fig. 6).

## Discussion

The data show that exposure of pregnant ewes in late gestation to chronic hypoxia in isobaric chambers led to sustained reductions in fetal $P_{aO_{2}}$ to mean levels of *ca* 11 mmHg and therefore similar to those measured in human infants of hypoxic IUGR pregnancy (Hecher *et al*. 1995) whilst not affecting maternal food intake. Chronic fetal hypoxia of this magnitude was accompanied by sustained reductions in fetal P aC O2, progressive increases in fetal haematocrit and variable increases in fetal blood lactate levels. Chronically hypoxic fetuses showed an impaired ontogenic increase in arterial blood pressure and a delayed ontogenic fall in fetal heart rate with advancing gestation. Parallel recording of carotid and femoral blood flow revealed sustained increases during the period of chronic hypoxia in chronically hypoxic fetuses, which were greater than those measured in normoxic fetuses with advancing gestation. The ratio of oxygen and glucose delivery to the fetal carotid circulation relative to the femoral circulation increased significantly and progressively in the chronically hypoxic fetus. Basal plasma urate concentrations were higher in the fetus than in the mother and plasma urate increased significantly in the chronically hypoxic fetus. Conversely, basal plasma ascorbic acid concentrations were similar in the mother and fetus and plasma ascorbic acid increased to similar extents only in the maternal circulation in normoxic and hypoxic pregnancy. These data support the hypotheses tested that the fetal brain sparing response persists during significant chronic fetal hypoxia and that an increase in ROS in the fetal circulation is an involved mechanism.

Several compensatory responses to hypoxia are regulated at least in part by the hypoxia‐inducible factor (HIF) family of transcription factors (Semenza, 2004). These coordinate intracellular responses to hypoxia by regulating the expression of hundreds of genes, including erythropoietin or EPO. The increased expression of the glycoprotein erythropoietin leads to increased red blood cell production, which can be measured as an elevation in packed red cell volume or the haematocrit. The present data confirm that this level of chronic hypoxia led to significant activation of the HIF‐regulated gene product erythropoietin, as the fetal and to a lesser extent maternal haematocrit increased progressively during and immediately following the period of chronic hypoxia. Additional blood gas data in the present study show that chronic hypoxia was accompanied by significant and sustained hypocapnia in the fetal but not in the maternal circulation. Fetal hypocapnia could be due to a shift in the fetal oxidative metabolism, decreasing fetal oxygen consumption and thereby fetal CO_2_ production and/or faster clearance of CO_2_ from the fetal to the maternal circulation. The sustained elevation in fetal rather than maternal blood lactate concentration during chronic hypoxia in the present study indicates an increase in fetal anaerobic metabolism, as has been previously suggested during hypoxic pregnancy (Lueder *et al*. 1995; Thompson, 2003). Bacon *et al*. (1984) also reported changes in placental barrier thickness and/or blood flow in chronically hypoxic guinea pig pregnancy.

Several studies have reported ontogenic increases in fetal arterial blood pressure and fetal peripheral blood flow and decreases in fetal heart rate with advancing gestation in several species (Reeves *et al*. 1972; Boddy *et al*. 1974; Dawes *et al*. 1980; MacDonald *et al*. 1983; Kitanaka *et al*. 1989; Forhead *et al*. 2000; Giussani *et al*. 2005). The present data are the first to report ontogenic increases in carotid blood flow with advancing gestation in control fetal sheep. One previous study reported lower mean values for fetal arterial blood pressure in chronically hypoxic fetuses of placentally restricted pregnancies (Edwards *et al*. 1999). However, others using the same placentally restricted model or in hypoxic fetuses from ovine pregnancies exposed to mild chronic hypoxia have reported similar fetal blood pressure between control and experimental animals (Kitanaka *et al*. 1989; Danielson *et al*. 2005; Pulgar *et al*. 2006, 2007, 2009; Poudel *et al*. 2015). By comparison, elevated basal values and alterations in the developmental decline of fetal heart rate with advancing gestation have been more consistently reported for the chronically hypoxic sheep fetus (Kitanaka *et al*. 1989; Pulgar *et al*. 2006). These findings are in keeping with a sympathetic dominant influence on cardiovascular control in the chronically hypoxic fetus (Kitanaka *et al*. 1989; Edwards *et al*. 1999). That blood flow to the fetal cerebral vascular bed increases in a sustained manner in response to chronic hypoxia has been established for many years (Richardson *et al*. 1993; Richardson & Bocking, 1998). In contrast, it has been generally assumed but widely accepted that blood flow to the peripheral circulations is decreased in the chronically hypoxic fetus and that this sustained redistribution of blood flow away from the periphery contributes to the repeatedly reported asymmetric growth restriction in the chronically hypoxic fetus (see Barker, 1996; Giussani, 2016). Two studies support lower basal values for femoral (Poudel *et al*. 2015) and carcass (Kamitomo *et al*. 1993) blood flow in the chronically hypoxic fetus, with single time point measurements with microspheres or acute recordings of femoral blood flow for 2 h. In this paper, we report that continuous longitudinal measurement of fetal femoral blood flow reveals a sustained increase during chronic hypoxia, akin to the peripheral dilatator response to hypoxia in the adult individual or to the enhanced basal femoral blood flow in adult offspring of chronically hypoxic pregnancy (Coney & Marshall, 2010). However, when calculating the actual delivery of oxygen and glucose to regional circulations, the ratio of substrate delivery to the carotid relative to the femoral circulation in the fetus shows a progressive increase as the chronic hypoxia develops. The latter provides first‐hand evidence for persistent brain sparing and continued redistribution of oxygen delivery away from peripheral circulations and towards the brain in the chronically hypoxic fetus.

The use of *in vivo* models to address questions regarding ROS generation comes with complications, as free radicals, by their very nature, are difficult to measure in these preparations. This problem is further compounded in the present study due to relative inaccessibility of the fetus within the intrauterine environment within a hypoxic chamber. Nevertheless, of all the techniques available, dynamic changes in urate and ascorbate concentrations in plasma constitute two of the few accepted biomarkers of ROS generation within the circulation *in vivo* (Halliwell & Gutteridge, 2004). Plasma urate concentration is an established marker of the activation of the xanthine oxidase (XO) pathway and, hence, of superoxide anion (•O_2_^−^) generation (Berry & Hare, 2004). In sheep, ascorbate forms part of the endogenous antioxidant defence as ovine species possess the enzyme gulonolactone oxidase, which promotes the *de novo* synthesis of ascorbate via the hexuronic acid pathway of the liver and/or kidney (Banhegyi *et al*. 1997). It is established that plasma ascorbate concentrations also increase throughout gestation in several species, consistent with a functional role for this antioxidant in prenatal life (Kolb *et al*. 1991). We have previously reported the discovery that enhanced ROS generation contributes to the fetal peripheral vasoconstrictor response to an episode of acute hypoxia, part of the fetal brain sparing effect (Thakor *et al*. 2010, 2015; Kane *et al*. 2012, 2014). ROS do so by quenching NO and promoting a vascular oxidant tone that complements carotid chemoreflex and endocrine constrictor mechanisms, aiding the redistribution of blood flow away from peripheral circulations (Giussani, 2016). Data from the present study suggest that there may be tonic activation of the XO pathway during basal conditions in the fetus relative to the mother, and that the XO pathway in the fetus is more sensitive to chronic hypoxia than the mother. As basal $P_{aO_{2}}$ is about one‐quarter lower in the fetal than in the maternal arterial circulation, it is tempting to speculate that the XO pathway is more active during basal conditions and more responsive to chronic hypoxia in fetal than in adult life, purely by virtue of this difference in oxygenation (Everest *in utero*; Barcroft *et al*. 1933). The significant increase in plasma urate concentrations in the circulation of the chronically hypoxic fetus is consistent with sustained activation of the XO pathway and continued excess ROS generation. Sustained XO‐derived ROS generation may thus contribute to the vascular oxidant tone of the fetal peripheral circulations, aiding in the shift in the delivery of oxygenated blood away from the fetal femoral and towards the fetal cerebral circulation in chronically hypoxic pregnancy of this magnitude. However, it is also possible that differences in circulating urate concentrations between mother and fetus reflect, in part, different rates of protein degradation and/or differences in renal clearance in the ewe and offspring.

Historically, there have been seminal investigations which have induced chronic fetal hypoxia by impairing uteroplacental blood flow by carunclectomy (Robinson *et al*. 1979; Poudel *et al*. 2015), placental embolisation (Block *et al*. 1984; Boyle *et al*. 1984; Gagnon *et al*. 1997), restriction of uterine blood flow (Richardson & Bocking, 1998; Stein *et al*. 1999; Lang *et al*. 2000), single umbilical artery ligation (Oyama *et al*. 1992; Supramaniam *et al*. 2006) and umbilical cord compression (Itskovitz *et al*. 1987; Giussani *et al*. 1997). However, all of these experimental manipulations reduce nutrient as well as oxygen delivery to the fetal circulation, preventing elucidation of the effects of chronic hypoxia on fetal cardiovascular function in isolation. Other equally important contributions have included description of fetal or neonatal cardiovascular function at the conclusion of the chronic hypoxic exposure (Rouwet *et al*. 2002; Sharma *et al*. 2006; Herrera *et al*. 2008, 2012; Tintu *et al*. 2009; Camm *et al*. 2010; Lindgren & Altimiras 2013; Iversen *et al*. 2014). A cluster of studies has investigated the effects of chronic hypoxia on fetal cardiovascular function *in vivo*, but only reported effects on fetal arterial blood pressure, heart rate and ventricular output (Alonso *et al*. 1989; Kitanaka *et al*. 1989; Kamitomo *et al*. 1994; Pulgar *et al*. 2006, 2007, 2009; Tissot van Patot *et al*. 2012). Another significant series of investigations has exploited the natural hypobaric hypoxia of high altitude to study the effects on fetal cardiovascular function of long‐term hypoxic gestation (Kamitomo *et al*. 1992, 1993, 2002; Browne *et al*. 1997 *a*,*b*; Onishi *et al*. 2003). While these studies have provided highly important contributions to the field of knowledge, exposure of pregnant ewes to altitudes between 3000 and 4500 m above sea level yields late gestation fetal arterial $P_{O_{2}}$ levels between 15 and 19 mmHg (Kamitomo *et al*. 1992, 1993, 2002; Browne *et al*. 1997 *a*,*b*; Onishi *et al*. 2003; Tissot van Patot *et al*. 2012). These values are much milder than those measured in the umbilical cord of the human hypoxic fetus in IUGR pregnancy, which are closer to 10–12 mmHg (Hecher *et al*. 1995). Investigation of this level of significant chronic fetal hypoxia using high altitude would involve exposure at 6500–7000 m above sea level (Gallagher & Hackett, 2004). Therefore, in summary, the work presented has introduced to the field of study a new technique for physiological research able to maintain chronically instrumented maternal and fetal sheep for prolonged periods of gestation under significant and controlled isolated chronic fetal hypoxia beyond levels that can be achieved by habitable high altitude. This technology also permits real time wireless recording in free moving animals of *in vivo* continuous maternal and fetal cardiovascular function, including alterations in regional blood flow signals as the hypoxic pregnancy is developing. Bioethically, the technology not only improves the physiological quality of the maternal and fetal *in vivo* data but it also improves animal welfare. This is the first time that this has been possible.

## Additional Information

### Competing interests

DAG, EAH and ADK worked with Telstar ACE to design the hypoxic chambers and with Maastricht Instruments to design and create the data acquisition system.

### Author contributions

The experiments in this study were performed in the Department of Physiology, Development and Neuroscience, University of Cambridge. BJA, KLB, YN, ADK, EAH, AST, KJB, CMC, NI, KLS, CB and DAG conceived and designed the experiments. BJA, KLB, YN, ADK, EAH, AST, KJB, CMC, NI, KLS, CB and DAG collected, analysed and interpreted the experimental data. BJA, KLB, YN, ADK, EAH, AST, KJB, CMC, NI, KLS, CB and DAG drafted the article and revised it critically for important intellectual content.

### Disclosure

License agreement 100395 CamDAS: Technology for simultaneous wireless recording of arterial blood pressure and blood flow in large animals. Giussani, D.A., Maatricht Instruments and Cambridge Enterprise.
